# Inflammatory and Myocardial Biomarker Response Following Pulmonary Vein Isolation: Cryoballoon Versus Balloon‐in‐Basket Pulsed Field Ablation

**DOI:** 10.1111/jce.70221

**Published:** 2025-12-08

**Authors:** Jan‐Per Wenzel, Sascha Hatahet, Charlotte Eitel, Raed Abdessadok, Sorin Popescu, Julius Nikorowitsch, Suzanne de Waha, Tanja Zeller, Karl‐Heinz Kuck, Roland Richard Tilz

**Affiliations:** ^1^ Department of Rhythmology, University Heart Center Lübeck University Hospital Schleswig‐Holstein Lübeck Germany; ^2^ German Center for Cardiovascular Research (DZHK) Partner Site Lübeck Germany; ^3^ Institute for Cardiogenetics University Hospital Schleswig‐Holstein Lübeck Germany

**Keywords:** atrial fibrillation, catheter design, cryoballoon, inflammation, myocardial injury, platelets, pulmonary vein isolation, pulsed field ablation

## Abstract

**Background:**

Cryoballoon ablation (CB) is a well‐established thermal technique for pulmonary vein isolation (PVI), while balloon‐in‐basket pulsed field ablation (BiB‐PFA) represents a novel non‐thermal alternative. Both single‐shot systems may trigger systemic inflammation and myocardial injury, yet direct comparisons are lacking. This study aimed to compare inflammatory and myocardial biomarker responses following first‐time PVI using CB or BiB‐PFA.

**Methods:**

In this prospective, single‐center study, 100 patients undergoing PVI for symptomatic paroxysmal or persistent atrial fibrillation (AF) were enrolled (CB: *n* =  50; BiB‐PFA: *n* =  50). Venous blood samples were collected before and on the morning after ablation to assess leukocytes, C‐reactive protein (CRP), platelets, troponin T, creatine kinase (CK), myoglobin, creatinine, and estimated glomerular filtration rate. Baseline characteristics, procedural data, and acute success were analyzed.

**Results:**

Patients in the CB group were older (74 vs. 65 years; *p* =  0.01) and had higher CHA₂DS₂‐VAcore (3.0 vs. 2.0; *p*  =  0.009). Acute PVI was achieved in all cases CB was associated with greater increases in leukocytes (Δ2.5 vs. 1.1 × 10⁹/L; *p*  =  0.05) and CRP (Δ5.8 vs. 3.4 mg/L; *p* =  0.02), whereas BiB‐PFA showed higher rises in CK (Δ217 vs. 103 U/L; *p*  =  0.01) and troponin T (Δ1129 vs. 614.5 ng/L; *p* =  0.01). No significant correlation was found between energy delivery and biomarker changes.

**Conclusion:**

CB and BiB‐PFA elicit distinct systemic responses. CB provoked stronger inflammatory activation, while BiB‐PFA caused greater myocardial biomarker release, suggesting energy‐ and device‐specific effects.

## Graphical abstract

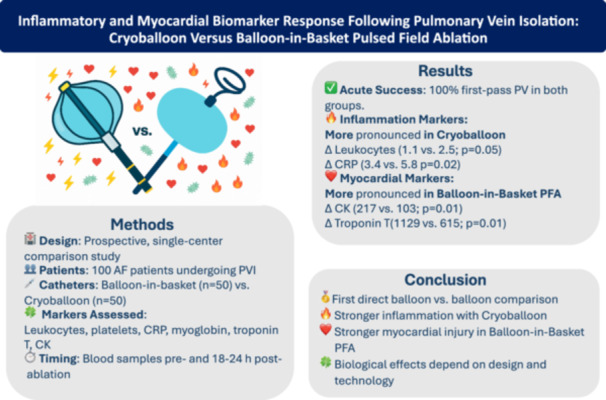

## 1 Introduction

Single‐shot ablation technologies have become widely adopted for pulmonary vein isolation (PVI) in patients with atrial fibrillation (AF), offering procedural efficiency and reproducible lesion formation. Among these, the cryoballoon (CB) system is the most established, delivering circumferential lesions via cryothermal energy [1]. In contrast, pulsed field ablation (PFA) has recently emerged as a non‐thermal modality utilizing irreversible electroporation. The balloon‐in‐basket (BiB) catheter (VOLT, Abbott) represents a novel PFA system that combines balloon stability with circumferential electrode deployment [2].

While both systems have demonstrated high acute efficacy and safety [1, 2], their systemic biological effects remain incompletely characterized. CB ablation is known to induce systemic inflammation and myocardial injury, as reflected by postprocedural increases in biomarkers such as C‐reactive protein (CRP), leukocyte count, troponin T, and creatine kinase (CK) [3]. Emerging evidence suggests that even non‐thermal PFA may provoke similar systemic responses, despite its tissue‐selective mechanism of action [4].

Systemic biomarker profiling post‐ablation provides insights into the acute biological impact of different energy sources and may inform catheter selection and post‐procedural management. This study aimed to compare inflammatory and myocardial biomarker dynamics following PVI using CB versus BiB‐PFA in patients with AF.

## 2 Methods

### 2.1 Study Population and Trial Design

**Figure 1:**
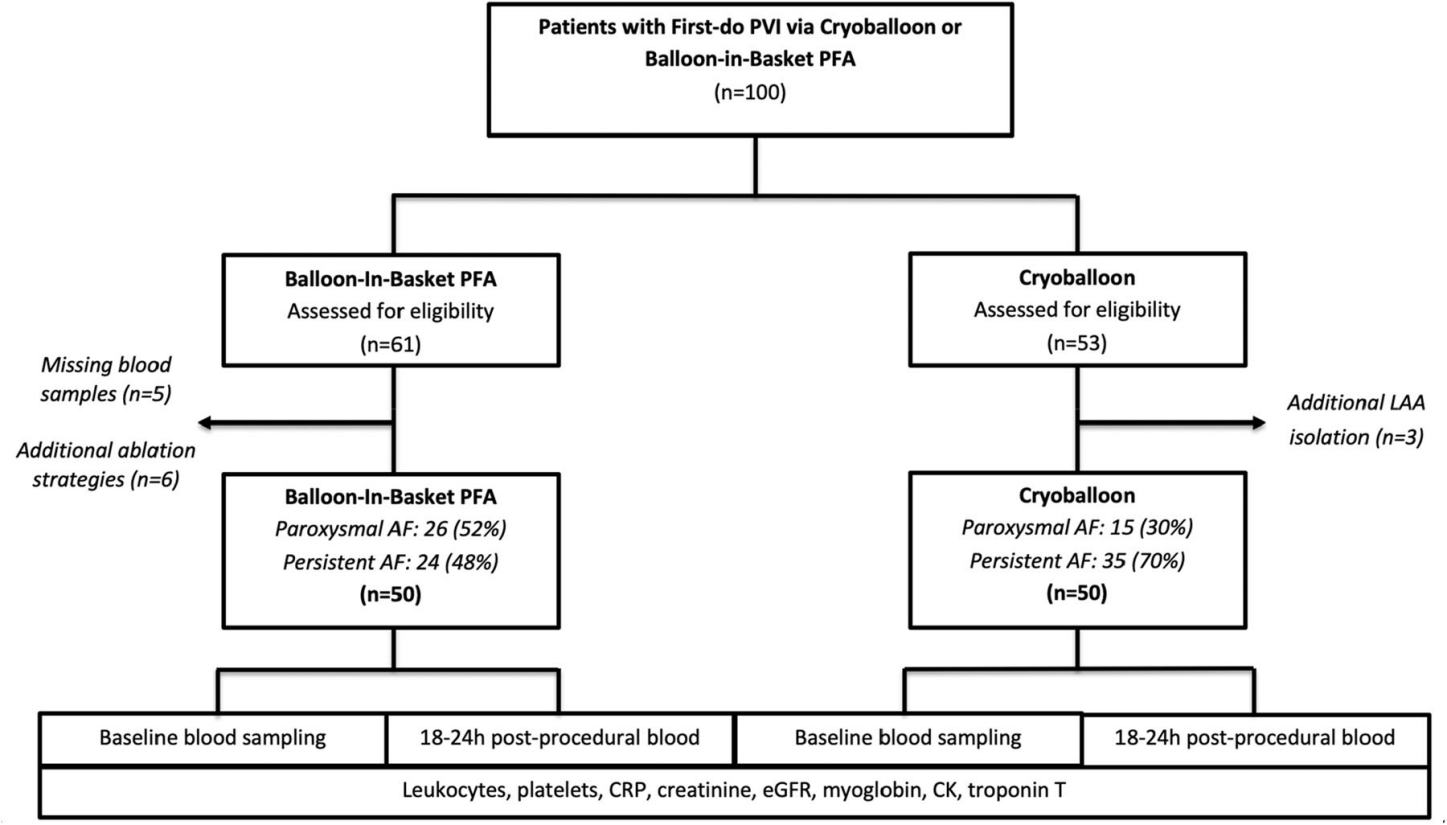
Study PRISMA (STROBE format). represents the study flow chart. Abbreviations: AF = atrial fibrillation, CK = creatine kinase, CRP = C‐reactive protein, eGFR = estimated glomerular filtration rate, LAA = left atrial appendage, PFA = pulsed field ablation, PVI = pulmonary vein isolation.

This prospective, single‐center, non‐randomized study investigated systemic biomarker changes following PVI using two single‐shot ablation modalities: CB and BiB‐PFA. Between January 2024 and July 2025, consecutive patients with paroxysmal or persistent AF were prospectively enrolled. All procedures were performed at the Heart Center Lübeck and documented in the institutional Ablation Registry (Figure 1). A subset of patients treated with BiB‐PFA (*n* = 27) also participated in the VOLT CE Mark study.

Eligible participants were ≥ 18 years of age with documented AF and provided written informed consent. To minimize potential confounding of biomarker measurements, exclusion criteria included active infection, autoimmune or chronic inflammatory disease, recent myocardial infarction, neuromuscular disorders, and significant hepatic impairment.

The study was approved by the local ethics committee (Lübeck Ablation Registry, approval number WF‐028/15) and conducted in accordance with the Declaration of Helsinki [5].

### 2.2 General Procedural Management

All patients underwent a standardized preprocedural evaluation in accordance with institutional protocols. In patients with elevated thromboembolic risk, transesophageal echocardiography was performed to exclude left atrial thrombus. Left ventricular ejection fraction (LVEF) and left atrial volume index were assessed in all patients.

Vitamin K antagonists were continued at therapeutic international normalized ratio levels (2.0–3.0), whereas direct oral anticoagulants were withheld on the morning of the procedure.

Ablation procedures were performed under deep sedation using propofol, midazolam, and fentanyl. In selected cases, continuous propofol infusion was omitted to maintain partial responsiveness. These cases were managed with a multimodal analgesic regimen including metamizole, midazolam, fentanyl, and lidocaine.

Vascular access was obtained via two ultrasound‐guided femoral venous punctures using 8 Fr sheaths. A diagnostic catheter was advanced into the coronary sinus, and transseptal puncture was performed under fluoroscopic guidance using the modified Brockenbrough technique. Following transseptal access, intravenous unfractionated heparin was administered to maintain an activated clotting time > 300 s. Left atrial access was established via an SL1 sheath (Abbott).

### 2.3 Cryoballoon Catheter

CB‐PVI was performed using either the POLARx or POLARx FIT (28 or 31 mm; Boston Scientific) or Arctic Front Advance Pro cryoballoon (28 mm; Medtronic). Both catheters were introduced through a dedicated steerable sheath, and all procedures followed a standardized ablation protocol. Pulmonary vein (PV) angiography was performed via SL1 sheath to visualize PV anatomy and guide balloon positioning. A time‐to‐isolation (TTI)‐guided ablation strategy was applied with a standard freeze duration of 180 s. If TTI exceeded 60 s, a bonus application was delivered. Phrenic nerve pacing and esophageal temperature monitoring were routinely performed. Acute PVI was confirmed by demonstrating entrance block using a circular mapping catheter.

### 2.4 Balloon‐in‐Basket Catheter

Procedures using the BiB‐PFA system were conducted according to the VOLT CE Mark protocol. In the first 30 patients, left atrial voltage mapping was performed using a high‐density mapping catheter. For subsequent cases, anatomical reconstruction was conducted with the BiB‐PFA catheter alone. After mapping, the transseptal sheath was exchanged for a steerable 13 Fr sheath (Agilis NxT, Abbott), and PV access was obtained using a 0.035‐inch guidewire.

PFA was delivered at 1800 V with a minimum of two rotated applications per vein. A maximum of eight applications per vein was allowed. For right‐sided PVs, phrenic nerve capture was assessed by spline pacing. If diaphragmatic stimulation was detected, ablation was continued at a reduced voltage of 1400 V with a minimum of three applications.

### 2.5 Postprocedural Management

Hemostasis was achieved using either vascular closure devices or figure‐of‐eight sutures combined with a compression bandage. The bandage was removed after 1–4 h, and sutures were taken out on the following day. Transthoracic echocardiography was routinely performed immediately after the procedure, at 1 h, and on the first postoperative day to exclude pericardial effusion. Oral anticoagulation was resumed 6 h after ablation and continued for a minimum of 2 months. Long‐term anticoagulation was guided by individual thromboembolic risk based on the CHA₂DS₂‐VA score, in accordance with current guideline recommendations [6, 7]. A class IC or III antiarrhythmic drug and a proton pump inhibitor were prescribed for 2 months.

### 2.6 Blood Sampling and Analysis

Venous blood samples were collected at two time points: (1) after femoral venous access prior to ablation, and (2) on the morning of the first post‐procedural day. All samples were obtained under fasting conditions. Analyzed biomarkers included leukocyte and platelet counts, CRP, high‐sensitivity troponin T, CK, myoglobin, serum creatinine, and estimated glomerular filtration rate (GFR). Leukocytes and platelet counts were measured from EDTA blood via fluorescence flow cytometry (Sysmex XN‐9000). CRP levels were assessed by immunoturbidimetry (Cobas c503). Troponin T and myoglobin were quantified using electrochemiluminescence immunoassay (Cobas 801). CK was measured enzymatically via UV photometry (Cobas c702), and serum creatinine via the Cobas c503. eGFR was calculated using the CKD‐EPI formula. All analyses were performed in the central laboratory of the University Hospital Schleswig‐Holstein using validated, certified protocols.

### 2.7 Statistical Analysis

Categorical variables are reported as absolute and relative frequencies and compared using Fisher's exact or *χ* ^2^ tests, depending on sample size. Continuous data were tested for normality using the Shapiro–Wilk test and are presented as median with interquartile range. Between‐group comparisons were analyzed using independent‐samples *t*‐tests or Mann–Whitney U tests. Effect sizes for within‐group were expressed as Hodges–Lehmann median differences with 95% confidence intervals. Within‐group comparisons were performed using paired *t*‐tests or Wilcoxon signed‐rank tests.

Correlations between biomarker changes and number of PFA applications were evaluated using Pearson or Spearman coefficients, based on data distribution. Two‐sided *p* values < 0.05 were considered statistically significant. To account for multiplicity, false discovery rate (FDR) control using the Benjamini–Hochberg procedure was applied to the inter‐group comparisons of Δ‐biomarker values. Multivariable linear regression was performed for each Δ‐biomarker to adjust for baseline and procedural variables, including age, CHA₂DS₂‐VA score, LVEF, AF type, procedure duration, contrast volume, and intraprocedural cardioversion, with results reported as β‐coefficients and *p* values. All analyses were performed using IBM SPSS Statistics v29.0.1.0 (IBM Corp., Armonk, NY, USA).

## 3 Results

### 3.1 Baseline Characteristics

A total of 100 patients were included (CB: *n *= 50; BiB‐PFA: *n* = 50). Baseline characteristics are displayed in Table 1. Patients in the CB group were older (74 vs. 65 years, *p* =  0.01) and had higher CHA₂DS₂‐VA scores (3.0 vs. 2.0, *p* =  0.02). Persistent AF was more prevalent in the CB group (70.0% vs. 48.0%, *p*  =  0.05). Sex, BMI, comorbidities, and LAVI were comparable between groups. LVEF was lower in the CB group (54.0% vs. 55.0%, *p*  =  0.01). At baseline, AF was present in 46.0% (CB) and 44.0% (BiB‐PFA, *p*  =  0.55).

**Table 1:** Baseline characteristics of the study population.

Variable	Total (*n* = 100)	BiB‐PFA group (*n* = 50)	CB group (*n* = 50)	*p* value
Age (years)	67 [62; 78]	65 [58; 71]	74 [63; 78]	0.01
Sex (female), *n* (%)	45 (45%)	24 (48%)	21 (42%)	0.68
BMI (kg/m²)	27.5 [24.6; 31.8]	27.1 [24.2; 28.7]	28.3 [24.6; 31.8]	0.24
AF type, *n* (%):				0.05
‐ Paroxysmal	41 (41%)	26 (52%)	15 (30%)	
‐ Persistent	59 (59%)	24 (48%)	35 (70%)	
Arterial hypertension, *n* (%)	62 (63%)	27 (54%)	36 (72%)	0.06
Diabetes mellitus, *n* (%)	15 (16%)	6 (12%)	9 (18%)	0.40
Coronary artery disease, *n* (%)	18 (18%)	7 (14%)	11 (22%)	0.30
Heart failure, *n* (%)	15 (15%)	4 (8%)	11 (22%)	0.09
Stroke/TIA, *n* (%)	7 (7%)	3 (6%)	4 (8%)	0.70
OSAS, *n* (%)	5 (5%)	2 (4%)	7 (14%)	0.08
CHA₂DS₂‐VA Score	3.0 [1.0; 3.0]	2.0 [0.0; 3.0]	3.0 [1.0; 3.8]	0.02
LAVI (mL/m²)	36.0 [33.8; 43.5]	33.0 [26.5; 47]	38.5 [33.8; 43.5]	0.51
LVEF (%)	55 [47; 55]	55 [55; 60]	54 [47; 55]	0.01
OAC, *n* (%)	84 (84%)	38 (76%)	46 (92%)	0.14
AF at procedure, *n* (%)	45 (45%)	22 (44%)	23 (46%)	0.55
NTproBNP (ng/L)	397 [96; 858]	212 [89; 971]	631 [130; 763]	0.16

*Note:* Values are presented as median [interquartile range], mean ± standard deviation, or number (percentage), as appropriate.

Abbreviations: AF = atrial fibrillation, BMI = body mass index, LAVI = left atrial volume index, LVEF = left ventricular ejection fraction, OAC = oral anticoagulation, OSAS = obstructive sleep apnea syndrome, TIA = transient ischemic attack.

### 3.2 Procedural Characteristics

Procedural details are shown in Table 2. Acute PVI was achieved in all patients. Procedure time was longer with BiB‐PFA (66.0 vs. 49.5 min, *p*  <  0.001), while dose‐area product was lower with (361 vs. 700 cGy·cm², *p*  <  0.001).

**Table 2:** Procedural characteristics of the study population.

Variable	Total (*n* = 100)	BiB‐PFA group (*n* = 50)	CB group (*n* = 50)	*p* value
Procedure time (min)	55.0 [43.0; 67.3]	66.0 [53.3; 73.8]	49.5 [42.3; 55.8]	< 0.001
Fluoroscopy time (min)	9.1 [6.5; 11.2]	8.5 [6.1; 10.5]	9.2 [7.2; 12.0]	0.40
Dose area product (cGy·cm²)	454 [301; 797]	361 [256; 514]	700 [375; 1200]	< 0.001
Contrast amount (mL)	50 [40; 60]	50 [40; 50] 45.2 ± 8.6	50 [40; 60] 54.1 ± 15.5	< 0.001
Successful acute PVI, *n* (%)	100 (100)	50 (100%)	50 (100%)	1.00
Intraprocedural cardioversion, *n* (%)	49 (49%)	16 (32%)	33 (66%)	< 0.001
Applications/cycles per subject, *n*		16 [14; 17]	4 [4; 5]	
Total freeze duration (min)			12 [12; 15]	
Major adverse events, *n* (%)		0 (0)	0 (0)	1

*Note:* Values are presented as median [interquartile range] or number (percentage), as appropriate.

Within the BiB‐PFA group, the first 30 procedures utilized high‐density voltage mapping, whereas the final 20 used anatomical reconstruction using the BiB‐PFA catheter alone. This transition was associated with reduction in both procedure time (71.5 vs. 38.5 min) and fluoroscopy time (9.3 vs. 6.8 min). Contrast use was lower in BiB‐PFA (*p* < 0.001). Cardioversion during ablation was more frequently performed in the CB group (66.0% vs. 32.0%, *p*  < 0.001). The number of applications was higher in BiB‐PFA compared to freeze cycles in CB (16.0 vs. 4.0, *p* < 0.001). No major complications occurred in either group (Table 2). Specifically, there were no cases of pericardial effusion or tamponade, stroke or transient ischemic attack, clinically apparent esophageal injury, acute kidney injury, or phrenic nerve palsy (neither transient nor persistent).

### 3.3 Biomarkers—Pre/Post Comparisons

Inflammatory and myocardial biomarkers changed significantly in both groups (Table 3). Leukocytes counts increased following ablation both the CB and BiB‐PFA groups (*p*  < 0.001). Platelets counts rose in the CB group (*p* <  0.001), whereas they declined in the BiB‐PFA group (*p*  < 0.001). CRP levels increased in both groups (*p*  < 0.001). Troponin T and CK rose in both cohorts (both *p* < 0.001). Myoglobin increased in CB (*p* = 0.001) and BiB‐PFA (*p* < 0.001). eGFR declined in CB (*p *= 0.03) but not in BiB‐PFA (*p*  = 0.49).

**Table 3:** Comparison of pre‐ and post‐procedural values for hemolysis and renal function markers in BiB‐PFA group and the CB group.

	Timing	Total (*n* = 100)	*p* value	BiB‐PFA group (*n* = 50)	*p* value	CB group\(*n* = 50)	*p* value
Leukocytes (*10^9/L)	Pre	6.1 [4.9; 7.3]	< 0.001	6.2 [5.2; 7.8]	< 0.001	5.8 [4.8; 6.7]	< 0.001
	Post	8.4 [7.1; 9.5]		8.3 [6.6; 9.3]		8.5 [7.3; 9.7]	
Platelets (*10^9/L)	Pre	201.0 [164.5; 237.5]	0.71	208.5 [172.8; 254.5]	< 0.001	194.0 [160.8; 238.0]	< 0.001
	Post	195.5 [160.5; 240.8]		195.5 [161.3; 243.0]		193.0 [175.3; 246.3]	
CRP (mg/L)	Pre	1.2 [0.6; 2.4]	< 0.001	1.1 [0.6; 2.0]	< 0.001	1.2 [0.6; 3.1]	< 0.001
	Post	6.9 [4.8; 12.0]		7.3 [3.7; 9.6]		6.8 [5.2; 14.3]	
Creatinine (µmol/L)	Pre	82.4 [74.2; 100.0]	0.41	87.1 ± 19.2	0.39	82.2 [77.6; 93.6]	0.23
	Post	87.0 [75.3; 99.8]		88.4 ± 21.7		86.7 [78.6; 96.0]	
eGFR(mL/min)	Pre	74.1 ± 19.3	0.03	77.0 ± 20.0	0.49	70.9 ± 18.8	0.03
	Post	71.7 ± 19.0		75.8 ± 18.7		68.2 ± 18.7	
Myoglobin (ng/mL)	Pre	42.0 [33.0; 56.0]	< 0.001	42.0 [32.5; 50.8]	< 0.001	40.5 [34.0; 60.3]	0.001
	Post	49 [41.0; 70.5]		49.0 [42.3; 71.0]		50.0 [40.0; 68.0]	
CK (U/L)	Pre Post	79.5 [54.8; 120.3] 232.5 [158.5; 334.3]	< 0.001	85.0 [68.5; 120.5] 316.0 [229.0; 398.0]	< 0.001	71.0 [53.0; 119.0] 182.5 [135.5; 257.5]	< 0.001
Troponin T (ng/L)	Pre Post	10.4 [7.3; 15.0] 769.0 [523.0; 1172.0]	< 0.001	8.9 [5.7; 12.2] 1077.0 [700.0; 1414.0]	< 0.001	11.4 [8.0; 15.1] 627 [451.8; 923.5]	< 0.001

*Note:* Values are presented as median [interquartile range] or mean ± standard deviation.

Abbreviations: CK = creatine kinase, CRP = C‐reactive protein, eGFR = estimated glomerular filtration rate.

### 3.4 Comparison of Delta Values

**Figure 2:**
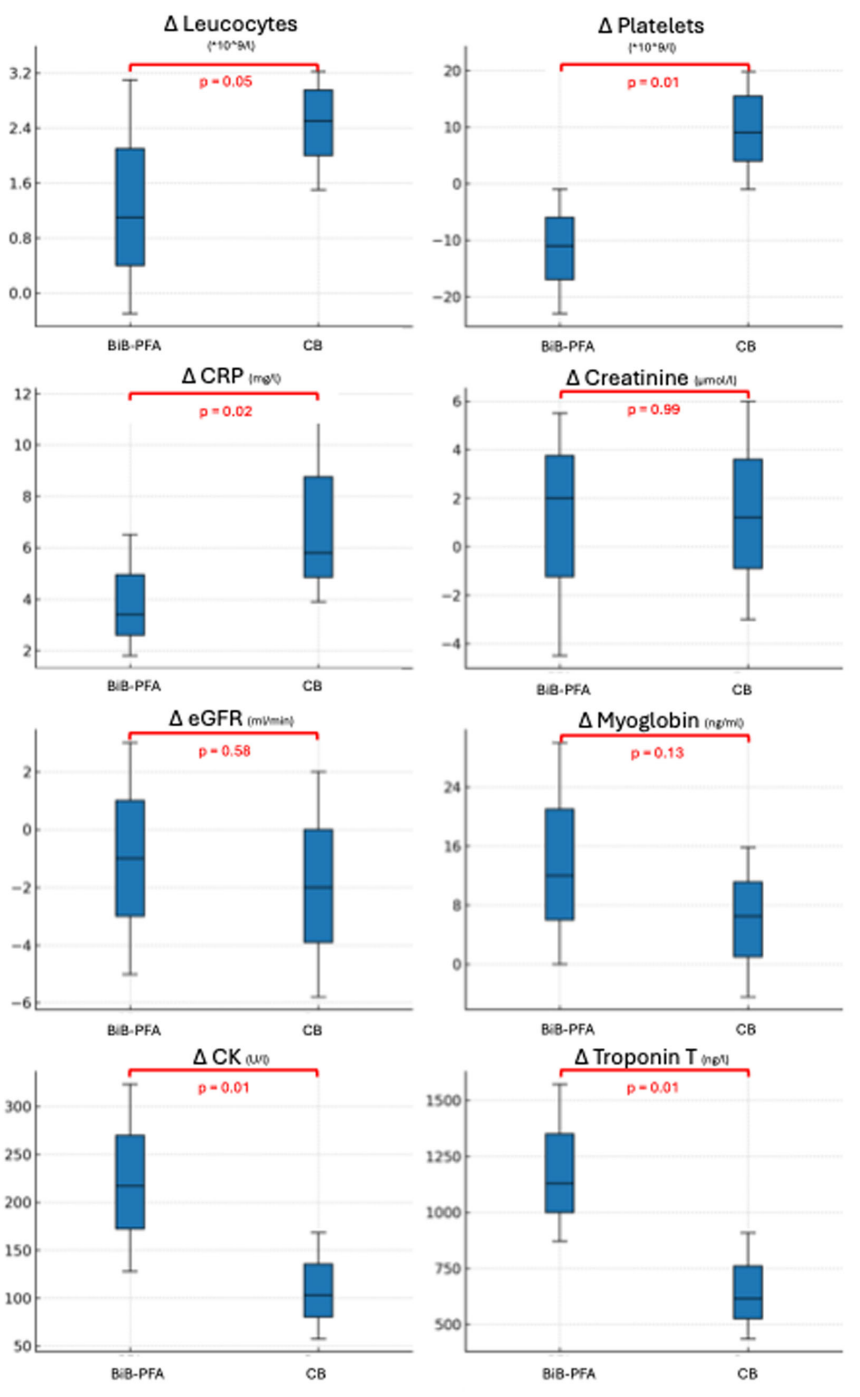
Comparison of delta values between the BiB‐PFA group and the CB group. Box plots represent the median and interquartile range (IQR) of changes from baseline to 18–24 h post‐procedure. Abbreviations: CK = creatine kinase; CRP = C‐reactive protein; eGFR = estimated glomerular filtration rate.

Intergroup comparison of delta values revealed significant differences in several biomarkers (Table 4). Leukocyte counts increased more markedly in the CB group (BiB‐PFA: +1.1 × 10⁹/L vs. CB: +2.5 × 10⁹/L, *p*= 0.05). Platelet counts demonstrated opposing trends, with a decrease observed in the BiB‐PFA group and an increase in the CB group (BiB: –11.0 × 10⁹/L vs. CB: +9.0 × 10⁹/L, *p* < 0.001). The rise in CRP was also more pronounced in the CB group (BiB‐PFA: +3.4 mg/L vs. CB: +5.8 mg/L, *p* = 0.02). In the BIB‐PFA group, troponin T rose to a greater extent compared to CB (BiB‐PFA: +1129 ng/L vs. CB: +614.5 ng/L, *p* = 0.01), as did CK (BiB‐PFA: +217 U/L vs. CB: +103 U/L, *p* = 0.01). Myoglobin levels also tended to increase more in the BiB‐PFA group, although this difference did not reach statistical significance (BiB‐PFA: +12.0 ng/mL vs. CB: +6.5 ng/mL, *p* = 0.13). No intergroup differences were observed for markers of renal function (Figure 2). Within the CB group, no difference according the delta values was observed (Table 5).

**Table 4:** Comparison of periprocedural changes (delta values) in inflammation and myocardial biomarkers between BiB‐PFA group and the CB group.

Variable	Total (*n* = 100)	BiB‐PFA group (*n* = 50)	CB group (*n* = 50)	Effect (HL, 95% CI)	*p* value
Δ Leucocytes (*10^9/L)	2.1 [0.5; 3.3]	1.1 [−0.3; 3.1]	2.5 [1.5; 3.4]	−1.2 (−2.1; −0.1)	0.05
Δ Platelets (*10^9/L)	−1.0 [−12.3; 17.3]	−11 [−23.0; −1]	9 [−1; 22]	−22.0 (−31.0; 13.0)	0.01
Δ CRP (mg/L)	5.2 [2.6; 5.2]	3.4 [1.8; 6.5]	5.8 [3.9; 11.7]	−2.5 (−4.4; −0.6)	0.02
Δ Creatinine (µmol/L)	1.4 [−3.9; 6.0]	2.0 [−4.5; 5.5]	1.2 [−3; 6]	0.0 (−3.5; 3.6)	0.99
Δ GFR (mL/min)	−1.5 [−5.0; 3.0]	−1.0 [−5.0; 3.0]	−2.0 [−5.8; 2]	1.0 (−2.0; 4.0)	0.58
Δ Myoglobin (ng/mL)	9.0 [−1.5; 24.0]	12.0 [0.0; 30.0]	6.5 [−4.5; 15.8]	7.0 (−1.0; 18.0)	0.13
Δ CK (U/L)	139 [83.5; 220]	217 [128.0; 323.0]	103 [57.5; 168.5]	99.0 (57.0; 159.0)	0.01
Δ Troponin T (ng/L)	859 [523; 1189]	1129.0 [870.8; 1571.0]	614.5 [435.1; 908.1]	514.2 (313.1; 734.8)	0.01

*Note:* Values are presented as mean and standard deviation or median [interquartile range]. Delta = post‐procedural minus pre‐procedural value.

Abbreviations: CI = confidence interval, CK = creatine kinase, CRP = C‐reactive protein, eGFR = estimated glomerular filtration rate, HL = Hodges‐Lehmann. Schätzer.

**Table 5:** Comparison of periprocedural changes (delta values) in inflammation and myocardial biomarkers within the CB group.

Variable	POLARx^TM^ (*n* = 20) − POLARx^TM^ (*n* = 5) − POLARx^TM^ FIT (*n* = 15)	Arctic front advance Pro^TM^ (*n* = 30)	*p* value
Δ Leucocytes (*10^9/L)	2.8 [2.2; 3.3]	2.2 [1.5; 3.6]	0.41
Δ Platelets (*10^9/L)	6.8 ± 17.6	12.2 ± 20.9	0.35
Δ CRP (mg/L)	7.7 [5.0; 12.6]	5.4 [2.2; 8.4]	0.10
Δ Creatinine (µmol/L)	1.7 [−2.3; 6.7]	−0.2 [−3; 4.8]	0.57
Δ GFR (mL/min)	−3 [−8.3; 0.3]	−0.5 [−3.8; 2.8]	0.15
Δ Myoglobin (ng/mL)	8.5 [2.8; 11.3]	4.5 [−9.8; 26.8]	0.87
Δ CK (U/L)	118 [90.8; 171.0]	83.5 [50.5; 162.0]	0.18
Δ Troponin T (ng/L)	690.0 [516.0; 967.0]	557.0 [406.0; 853.0]	0.18

*Note:* Values are presented as mean and standard deviation or median [interquartile range]. Delta = post‐procedural minus pre‐procedural value.

Abbreviations: CK = creatine kinase, CRP = C‐reactive protein, eGFR = estimated glomerular filtration rate.

### 3.5 Multivariable Linear Regression

Catheter type remained independently associated with Δ Leucocytes (*β* = −1.4, *p* = 0.06), Δ Platelets (*β* = −30.7, *p* < 0.001), Δ CRP (*β* = −8.0, *p* = 0.01), Δ Creatinine (*β* = 1.2, *p* = 0.70), Δ GFR (*β* = 2.2, *p* = 0.29), Δ Myoglobin (*β* = 11.8, *p* = 0.17), Δ CK (*β* = 113.9, *p* = 0.01), Δ Troponin‐T (*β* = 447.7, *p* < 0.001) in multivariable linear regression adjusting for age, CHA₂DS₂‐VA score, LVEF, AF type, procedural duration, amount of contrast media and intraprocedural cardioversion.

### 3.6 Correlation Between Energy Applications and Biomarker Changes

As shown in Table 6, no correlation was observed between the number of energy deliveries (freeze cycles in the CB group and PFA applications in the BiB‐PFA group) and changes in any of the evaluated biomarkers.

**Table 6:** Correlation between energy applications and biomarker changes.

Variable	BiB‐PFA group (*n* = 50)	*p* value	CB group (*n* = 50)	*p* value
Leucocytes	−0.062	0.69	0.096	0.51
Platelets	−0.042	0.78	−0.129	0.37
CRP	0.079	0.64	0.203	0.16
Creatinine	−0.101	0.52	0.083	0.56
GFR	0.171	0.27	0.006	0.97
Myoglobin	−0.246	0.14	0.138	0.40
CK	0.143	0.40	0.046	0.75
Troponin T	0.174	0.32	0.064	0.66

Abbreviations: CK = creatine kinase, CRP = C‐reactive protein, eGFR = estimated glomerular filtration rate.

## 4 Discussion

This prospective study compared inflammatory and myocardial biomarker responses following PVI using CB ablation versus BiB‐PFA system. The central findings can be summarized as follows: (i) inflammatory markers, particularly leukocytes and CRP, increased in both groups following ablation; (ii) Troponin T, CK, and myoglobin rose substantially in both cohorts; and (iii) intergroup comparison revealed greater increases in leukocytes and CRP in the CB group, while troponin T and CK elevations were more pronounced in BiB‐PFA.

### 4.1 Contrasting Injury Patterns: Apoptosis‐Driven Electroporation Versus Necrotic Thermal Lesions

PFA exhibits a striking dissociation between the extent of myocardial injury and the degree of systemic inflammation when compared to thermal ablation [4]. In this study, the BiB‐PFA system induced greater myocardial injury, as evidenced by higher post‐procedural troponin and CK levels, yet paradoxically triggered milder systemic inflammatory response with lower CRP and leukocyte increases than CB. This pattern aligns with emerging comparative studies, which have shown PFA to induce troponin elevations 1.6–1.9 times greater than both radiofrequency (RF) and CB [8], possibly reflecting more complete or extensive lesion formation, whereas inflammatory markers consistently appear lower after PFA than thermal ablation [4, 9]. While elevated myocardial injury markers imply extensive myocardial involvement with PFA, the clinical relevance remains uncertain. It is unclear whether these biomarker elevations represent irreversible electroporation or merely transient membrane disruption [4, 10, 11, 12, 13]. Future studies incorporating imaging and tissue characterization are needed to determine the permanence and physiological implications of PFA‐induced lesions.

Mechanistically, this divergence in biomarker response likely reflects fundamental differences in the mode of tissue injury. Thermal ablation causes coagulative necrosis via protein denaturation and membrane disruption, leading to extensive release of damage‐associated molecular patterns (DAMPs) and pronounced inflammatory activation [9, 14]. In contrast, PFA uses high‐intensity electric fields to induce irreversible electroporation, preferentially targeting cardiomyocytes while sparing the extracellular matrix and adjacent non‐myocardial cells [15]. The mode of cell death in PFA lesions is predominantly apoptotic rather than necrotic. Apoptosis is a programmed, contained form of cell demise that causes less spillage of intracellular contents and incites minimal inflammatory cell infiltration [16]. Thus, even though PFA creates extensive myocardial lesions, the apoptosis‐driven injury triggers only a mild systemic immune response [9, 16]. In simpler terms, the nature and context of cell death, and not merely its extent, mainly influence the magnitude of inflammation. Necrotic thermal injury provokes a stronger inflammatory systemic response than the more targeted, controlled injury of PFA.

### 4.2 Role of Collateral Damage in Inflammation

The milder inflammatory response observed with PFA likely also reflects reduced collateral tissue injury. Unlike CB, which can affect surrounding structures, PFA's tissue selectivity limits unintended damage to adjacent tissues and organs such as the esophagus, nerves, or pericardium, which are key contributors to systemic inflammation. This interpretation is supported by prior studies reporting lower rates of esophageal lesions and phrenic nerve palsy with PFA compared to thermal ablation [17]. Moreover, systemic inflammatory biomarkers appear to correlate more strongly with collateral tissue involvement than with the extent of myocardial injury itself [9]. In our study, CRP rose more markedly in the CB group despite lower myocardial enzyme release, supporting the hypothesis that inflammatory activation primarily arises from non‐cardiac tissue disruption. By sparing these structures, PFA may limit systemic immune activation even in the presence of extensive atrial lesions. Interestingly, platelet counts decreased in the BiB‐PFA group but increased following CB, a pattern that may reflect modality‐specific differences in shear stress, endothelial activation, and subsequent platelet consumption.

### 4.3 Catheter Design and Procedural Factors

Beyond energy modality, catheter architecture may also influence biomarker profiles. The BiB‐PFA system features a balloon‐in‐basket design with multiple splines that enable simultaneous mapping and ablation. One unique advantage is the real‐time visualization of electrode–tissue contact via electrogram signals directly from the splines [2]. This immediate feedback allows operators to identify suboptimal wall contact before energy delivery, enabling precise adjustment for improved lesion formation. In contrast, CB positioning relies primarily on fluoroscopic guidance using contrast media injection, which may delay the recognition of incomplete vein occlusion or suboptimal apposition. These technical differences may have multiple implications. First, the ability to confirm optimal contact in real time may lead to more consistent lesion formation and more effective pulmonary vein occlusion, potentially contributing to the higher myocardial biomarker release observed in BiB‐PFA and increased inflammation markers in CB PVI. Second, radiation exposure and contrast use can be reduced, enhancing procedural safety for both patient and operator. This is particularly relevant in anatomically challenging veins, where fluoroscopy‐based positioning may require multiple angiograms. Taken together, the integrated mapping and tissue contact assessment offered by the BiB‐PFA system may improve both procedural efficacy and efficiency while preserving a favorable biomarker profile. From a clinical perspective, it is important to consider whether these biological differences have early safety implications. In our cohort, acute procedural success was 100% in both groups, and no major periprocedural complications were observed. Specifically, there were no cases of pericardial effusion, stroke/TIA, esophageal injury, or transient and persistent phrenic nerve palsy. Thus, the observed divergence in biomarker release profiles did not translate into early clinically apparent adverse events. Larger, multicenter studies with sufficient statistical power are needed to validate these findings and to further explore potential links between acute biomarker release patterns and subsequent clinical outcomes.

Interestingly, we observed no significant correlation between the number of energy applications and biomarker changes in either group. This suggests that acute biological responses are not directly related to the absolute number of applications or freeze cycles, but may instead reflect lesion quality and the underlying mechanism of injury. In PFA, energy delivery functions more as a binary process, where electroporation is either successfully achieved or not. In contrast, thermal ablation is believed to produce cumulative tissue damage, with additional freeze cycles potentially intensifying collateral tissue damage. However, our data did not demonstrate a dose–response relationship for either modality, suggesting that tissue specificity and catheter–tissue interaction may play a more decisive role in shaping systemic biological responses than the number of applications alone.

### 4.4 Limitations

This study has limitations. First, it was conducted in a non‐randomized design at a single high‐volume center with a limited sample size, thereby restricting generalizability and limiting statistical power. Second, biomarkers were obtained only once after the procedure, the following morning, which precludes assessment of temporal dynamics such as onset, peak timing, and resolution of inflammatory and myocardial injury responses. Future studies should therefore incorporate multiple sampling time points (e.g., 6, 24, and 48 h) and precisely document the time interval between ablation completion and blood draw to allow evaluation of biomarker kinetics. Third, heterogeneity in anesthesia regimens may have influenced systemic inflammatory responses and introduced variability in biomarker interpretation. In addition, future investigations should include a broader biomarker panel, including inflammatory mediators and markers distinguishing apoptosis from necrosis, in order to further elucidate underlying biological pathways. Fourth, myocardial injury was inferred from biomarkers without imaging confirmation. Fifth, the CB and BiB‐PFA systems differed in catheter design, mapping integration, and operator experience, which may have introduced procedural variability. Sixth, significant differences were present in baseline characteristics between groups, particularly regarding age, LVEF, and CHA₂DS₂‐VA score, which may have influenced biomarker responses. Finally, most BiB‐PFA cases were enrolled in the VOLT CE Mark study, which may have introduced protocol‐related bias.

## 5 Conclusion

Both CB and BiB‐PFA systems were associated with significant systemic inflammatory and myocardial biomarker changes following PVI. While CB resulted in more pronounced inflammatory responses, BiB‐PFA was associated with greater myocardial injury. These findings suggest that, despite both systems employing balloon‐based designs, the underlying energy modality and catheter characteristics influence the biological impact of ablation. Further studies integrating imaging and longitudinal biomarker profiling are needed to clarify the clinical relevance of these divergent profiles.

## Conflicts of Interest

J.‐P.W.: funding (German Foundation of Heart Research F/29/19), speaker (Abbott, Doctrina Med), travel (Boston Scientific). C.E.: research/travel grants and speaker (Abbott, Boston Scientific, Lifetech, Biosense Webster, Cardiofocus, C.T.I. GmbH, Doctrina Med). S.P.: travel/congress grants (Lifetech), educational/speaker grants (Abbott), consulting (Active Health). J.N.: speaker (Abbott). S.d.W.: consulting (Zoll/Therox), speaker (Edwards Lifesciences). K.H.K.: grants/personal fees (Abbott Vascular, Medtronic, Biosense Webster). R.T.: speaker (Pfizer, Abbott, Biosense Webster, Boston Scientific, Doctrina Med, cme4u, Medtronic, Radcliffe, Wikonect), consulting/advisory (Boston Scientific, Biosense Webster, Capvision, Guidepoint, Haemonetics, Medtronic, Philips, Abbott), institutional research (Biotronik, Abbott, Boston Scientific, Medtronic, Lifetech, J&J), travel (Biosense Webster, Abbott, Boston Scientific, Medtronic, Philips).
